# Modulation Classification of Underwater Communication with Deep Learning Network

**DOI:** 10.1155/2019/8039632

**Published:** 2019-04-01

**Authors:** Yan Wang, Hao Zhang, Zhanliang Sang, Lingwei Xu, Conghui Cao, T. Aaron Gulliver

**Affiliations:** ^1^Department of Electrical Engineering, Ocean University of China, Qingdao 266100, China; ^2^School of Physics and Electronic Engineering, Taishan University, No. 525 Dongyue Street, Tai'an City, China; ^3^Technical Engineering Department of CRRC Qingdao Sifang Co., Ltd., Qingdao 266111, China; ^4^Department of Information Science Technology, Qingdao University of Science and Technology, Qingdao 266061, China; ^5^Department of Electrical and Computer Engineering, University of Victoria, Victoria, BC, Canada V8W 2Y2

## Abstract

Automatic modulation recognition has successfully used various machine learning methods and achieved certain results. As a subarea of machine learning, deep learning has made great progress in recent years and has made remarkable progress in the field of image and language processing. Deep learning requires a large amount of data support. As a communication field with a large amount of data, there is an inherent advantage of applying deep learning. However, the extensive application of deep learning in the field of communication has not yet been fully developed, especially in underwater acoustic communication. In this paper, we mainly discuss the modulation recognition process which is an important part of communication process by using the deep learning method. Different from the common machine learning methods that require feature extraction, the deep learning method does not require feature extraction and obtains more effects than common machine learning.

## 1. Introduction

Underwater acoustic communication is considered to be the most challenging wireless communication method [1]. The underwater acoustic channel makes communication difficult due to its own characteristics, such as narrow bandwidth, prolonged time, and serious intersymbol interference (ISI). These characteristics seriously affect the stability of the communication system and cause obstacles to high-rate underwater acoustic communication [2].

Modulation classification plays a decisive role in communication systems. At the communication receiver, signal demodulation and signal identification are based on correct modulation classification. Due to the complexity and instability of underwater acoustic communication systems, it is difficult to identify modulation during actual communication. There are now some methods of underwater acoustic communication that have been used, such as sparse adaptive convolution cores [3–5], time-domain turbo equalization [6, 7], and frequency-domain turbo equalization [8], but these methods still have the problem of high computational complexity and low classification success rate. To recognize modulation, accurately is hence becoming more challenging.

The traditional modulation classification method needs to extract the signal features first and then further classify and identify. With the improvement of modulation classification requirements in underwater acoustic communication, the modulation classification method is expected to adapt to the characteristics of the underwater acoustic communication system. These all lead to the need to consider new modulation identification methods via deep neural networks (DNNs) in underwater acoustic communication.

Deep learning (DL) is a new machine learning (ML) methodology that has found successful implementations in many application domains. Especially in the last period of time, this approach has become popular due to its state-of-the-art capability for big data analysis and processing ability [9]. As one of the most powerful classification tools, it has been applied in various application fields, such as computer vision [10], natural language processing [11], and speaker recognition [12]. Deep learning enables multilevel automatic feature representation learning. In contrast, traditional machine learning-based natural language processing (NLP) systems liaise heavily on hand-crafted features. Deep learning models have crushed other classical models on the task of image classification in a similar way. Especially in the last two years, most of the industrial products for speech recognition incorporated deep neural networks, and this success spurred a new wave of research into deep learning algorithms and architectures for automatic speech recognition (ASR), which is still ongoing today.

DL relies on massive amount of data and, for research and applications, this can be easily available in communications systems. Furthermore, unlike ML, DL has the advantage of not requiring manual feature selections and improves the classification rate greatly [13]. Recently, DNN method has been introduced to the field of communications, such as detection for communication systems using convolutional neural network (CNN) [14], orthogonal frequency-division multiplexing (OFDM) signal detection with DNN [15], and multi-input multi-output (MIMO) channel estimation [16].

At present, DNN has been widely used in image recognition and voice processing; especially, CNN has shown a strong role in these fields. CNN achieves recognition accuracy that surpasses other algorithms through a multilayered network structure, demonstrating its superiority in feature recognition. From a neurological perspective, the design of the convolutional neural network is inspired by the perception of the external world by the human brain's visual cortex. The human eye passes the perceived external things to the brain in the form of images. The brain abstracts the image layer by layer, extracting the edges of the image and other high-latitude features representing the image to make accurate judgments on the brain. The human eye transmits the perceived external things to the brain in the form of images. The brain passes through the layers of the image. Abstracting, extracting the corners of an image, represents the high-latitude features of the image to make accurate judgments for the brain.

In 1982, the original model neural sensor of the convolutional neural network [17] was proposed. In 1998, one of the common models LeNet for deep learning [18] was proposed. AlexNet won the ImageNet competition in 2012 [19]. Since then, based on AlexNet evolved a variety of forms of CNN, such as GoogleNet (Google network) [20], VGGNet (visual geometry group 3 network) [21], ResNet (residual network) [22], and DenseNet (densely connected convolutional networks) [23]. The rapid development of CNN-based image classification, speech, computational vision, and NLP technologies has now been achieved.

The content structure of the paper is as follows: Section 2 explains the model of the AMC (automatic modulation classification) system. In this section, we elaborate on the signal model that needs to be dealt with.  Section 3 introduces the modulation classification of the DL network structure. In the absence of feature extraction, modulation is directly identified by DL network. The simulation verifies the effectiveness of DL network in modulation classification through the underwater acoustic communication in Section 4. The final summary is in Section 5.

## 2. System Model

### 2.1. Signal Model

AMC is a necessary part of signal classification and demodulation at the receiver. Different from other commonly used machine learning methods, when using the deep learning method for modulation classification, this feature is not required for feature extraction. Without affecting the classification effect, the process is optimized and system efficiency is improved.

**Figure 1:**
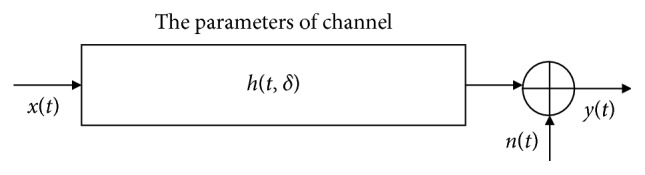
The underwater acoustic channel model.

We focus on a channel model based on the three factors: multipath fading, Doppler effect, and Gaussian noise. This channel model can be thought of as a convolution core with additive noise, as is shown in Figure 1 [24]. For ease of analysis, we define the received signal model as

(1) $$\begin{array}{c} y\left(t\right)=h\left(t,\delta \right)\;⊗\;x\left(t\right)+n\left(t\right)=\overset{N}{\underset{i=1}{\sum }}\alpha_{i}\left(t\right)x\left(t-\delta \left(t\right)\right)+n\left(t\right), \end{array}$$

where *x*(*t*) is the modulated transmission signal and *y*(*t*) is the received signal after interference with underwater acoustic channels and additive noise. Taking into account the complexity of the underwater acoustic channel, in order to better analyze the impact on the received signal, we use *h*(*t*, *δ*) to represent the underwater acoustic channel model parameters. *α*_*i*_(*t*) represents the attenuation coefficient. *δ*(*t*) represents the random time delay. *N* represents the number of multipaths. Additive noise *n*(*t*) assumes that statistical properties satisfy Gaussianity. The operator ⊗ means convolution.

### 2.2. Modulation Model

The commonly used modulation methods in underwater acoustic communication are MPSK (BPSK, QPSK, and 8PSK) and MQAM [6].

MPSK is the most commonly used modulation method. Phase shift is based on the phase as a variable, and the amplitude and the frequency are as a constant signal modulation. The MPSK signal can be represented by such a set of signals:

(2) $$\begin{array}{c} x_{\text{MPSK}}\left(t\right)=A\;\;\cos\left(w_{c}t+\theta_{m}\right), \end{array}$$

where *A* represents the amplitude, *w*_*c*_ represents the angular frequency, and phase *θ*_*m*_ is represented by a uniformly spaced set of phase angles.

(3) $$\begin{array}{c} \theta_{m}=\frac{2\left(m-1\right)\pi }{M}\text{,}\;\;m=1,2,\;\ldots ,M, \end{array}$$

where *M* represents the number of symbols and the phase interval between two adjacent signals in the modulation signal is 2*π*/*M*. For example, the phase spacing of the four symbols QPSK is *π*/2.

The expression of the MQAM signal is somewhat different from that of MPSK. It is expressed as

(4) $$\begin{array}{c} x_{\text{MQAM}}\left(t\right)=A_{i}\cos\left(w_{c}t\right)+B_{i}\sin\left(w_{c}t\right),\;i=1,2,\ldots ,M, \end{array}$$

where *A*_*i*_=*a*_*i*_cos(*ϕ*_*i*_) and *B*_*i*_=*b*_*i*_sin(*ϕ*_*i*_), respectively; they modulate two different carriers in MQAM modulation, in which *a*_*i*_ and *b*_*i*_ represent two sequences that need to be sent.

## 3. Technical Approach

**Figure 2:**
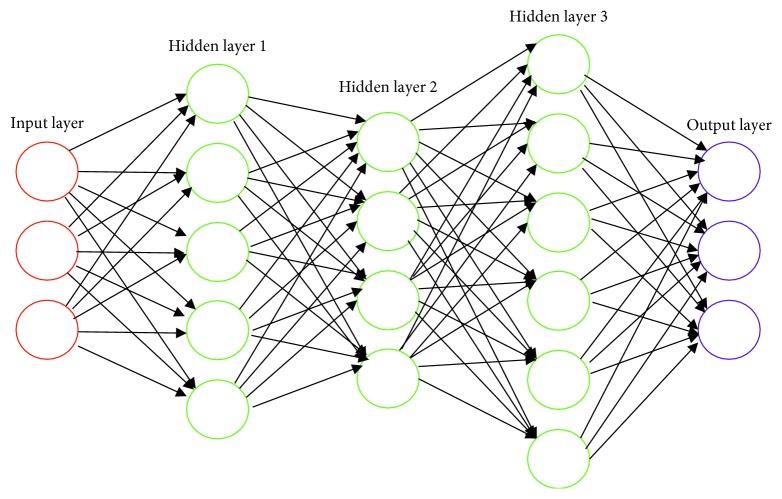
Simplified neural network diagram.

Previous studies have shown that a neural network with at least one hidden layer is a universal approximation [25]. As long as the level of the network is increased, any continuous function can be approximated. A simple neural network, shown in Figure 2, consists of an input layer, two hidden layers, and an output layer. The hidden layer in the middle can consist of more than 3 layers of neurons, and the number of neurons in each layer can be set to any number based on the actual objects to be fitted. In general, the number of layers in the neural network is not included in the input layer, and the number of hidden layers from the first layer to the output layer is the number of layers in the neural network. In theory, any function can be fitted if there are enough hidden layers and large enough datasets. However, in practical applications, too many hidden layers may result in overfitting. In other words, the neural network trained with training set data is very effective, but the effect is very poor in practical application. It is shown that the fitting function can only be useful for training data and the generality is poor.

Therefore, ideally, as long as the network is not overfit, the deeper the neural network should be the better. However, in actual situations, when the level of the neural network is continuously added, there will be an accuracy degradation problem. That is, the accuracy rate will rise first and then reach saturation. Continuously increasing the level will lead to a decrease in accuracy. This is not an overfit problem because not only does the error increase in the test set but also the error in the training set itself increases. When the network has a large number of levels, as the forward propagation proceeds, some information of the input data may be lost. The activation function and random deactivation is included in each layer, resulting in a general expression capability of the model.

**Figure 3:**
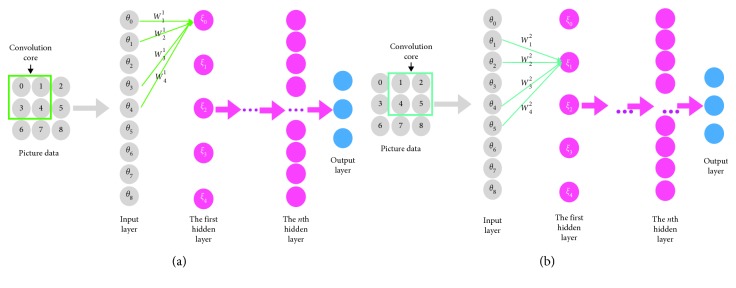
CNN simple illustration diagram. (a) Starting position and (b) move position.

From the development trend of the CNN structure, we use the convolutional layer of small convolution core size and pooling to obtain more data information and use multiple layers of fully connected layers to improve classification. In Figure 3, picture data are taken as an example for a simple explanation of common terms used in CNN. The convolution core size refers to the number of picture data that is framed. After the data enter the input layer by the convolution operation which become the first convolution layer 0 neuron's value, this process is equivalent to extract the image data information. In image data, the location of the convolution core from the left to the right is called stride, where the stride is 1, and you can set a value greater than 1 according to the situation.

The ordinal number of the image data corresponds to the data ordinal of the input layer in Figure 3, and the line from the input layer to the hidden layer is equivalent to a general convolution operation. For example, the convolution operation in the initial place of Figure 3 is expressed as

(5) $$\begin{array}{c} \xi_{0}=W_{1}^{1}\theta_{0}+W_{2}^{1}\theta_{1}+W_{3}^{1}\theta_{4}+W_{4}^{1}\theta_{5}, \end{array}$$

where *W*_*i*_^*j*^ represents weight, *i* represents the size of the filter, *i*=0, 1,…, 4, and *j* represents the serial number of stride, and there are two steps of convolution here, *j*=1,  2; *θ*_*ρ*_ represents the neurons of the input layer, *ρ*=0,  1,…,  8; *ξ*_*ℓ*_ represents the result of the first hidden layer computed through the input layer convolution; and *ℓ* represents the number of neurons in this layer, *ℓ*=0,  1,…,  4.

In the same way, the convolution operation in the move place of Figure 3 is expressed as

(6) $$\begin{array}{c} \xi_{1}=W_{1}^{2}\theta_{1}+W_{2}^{2}\theta_{2}+W_{3}^{2}\theta_{5}+W_{4}^{2}\theta_{6}. \end{array}$$

**Figure 4:**
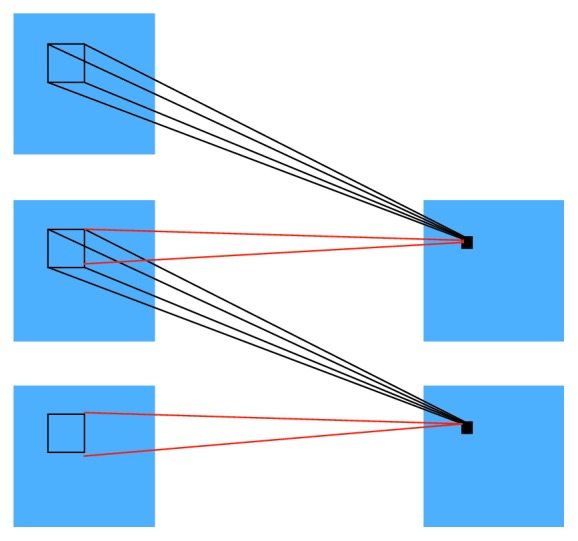
The 2D convolution operation.

A general convolution operations include 1D, 2D, and 3D operations. In 2D operation, for example, Figure 4 is a 2D CNN convolution operation for image sequences using 2D convolution cores. The temporal dimension of the convolution operation is 2; that is, a convolution operation is performed on two consecutive frames of data. The 2D convolution is to form a flat by stacking a plurality of consecutive frames, and then, a 2D convolution kernel is used in the flat. In this structure, each feature map in the convolutional layer is connected to multiple adjacent consecutive frames in the previous layer, thus capturing information. In Figure 4 to the left, the value of a position in a convolutional map is obtained by convolving local receptive fields at the same position in two consecutive frames one level above. The 2D convolution kernel can only extract one type of feature from the flat because the weights of the convolution kernels are the same in the entire flat. That is, the shared weights are the same convolution kernel (the same one in the figure, the connecting lines of the colors represent the same weights). A variety of convolution kernels can be used to extract a variety of features. 1D is used in NLP, and 2D and 3D convolutions are used for images. Among them, 2D convolution processes an image and 3D convolution processes multiple images. Contrast with 2D, 3D convolution considers the information of the time dimension.

The activation function determines whether a neuron is activated, whether the information received by the neuron is useful, and whether it should be left or discarded [26]. The nonlinear transformation of activation function can fit various kinds of classification curves so that the neural network can deal with very complicated tasks. As the number of layers of the neural network increases, the ability to fit the training dataset becomes stronger, which may result in overfitting. In order to avoid the above problems, this paper chooses a shallow network architecture and random deactivation technology in the CNN structure design to prevent overfitting. Random deactivation means using dropout technology to prevent overfitting by randomly discarding part of the data. The random deactivation here is dropout technology. After the application of dropout, some neurons will be inactivated and not connected with other neurons, so the phenomenon of overfitting can be reduced effectively. The inactivation here is random, meaning that the next time dropouting the inactivated neuron may be turned back on.

### 3.1. DL Architecture

The current DL architecture evolved from the VGGNet structure. The main reason VGGNet achieved good results at the ImageNet competition in 2014 was due to demonstrating the depth of the network is a key part of the algorithm's performance. In the DL architecture, we have designed for modulation classification that uses a similar structure, but it is different from the commonly used structure. The architecture we use contains four layers of convolution and three layers of full connecting layer.

The general CNN structure contains the pooling layer, and we also add this layer to the DL structure we designed. The performance of pooled operations is still useful for designing convolutional networks. The role of pooling operations in reducing parameters, controlling overfitting, improving model performance, and saving computational power is still evident. Therefore, the pooling operation is an indispensable operation in the convolution design.

**Figure 5:**
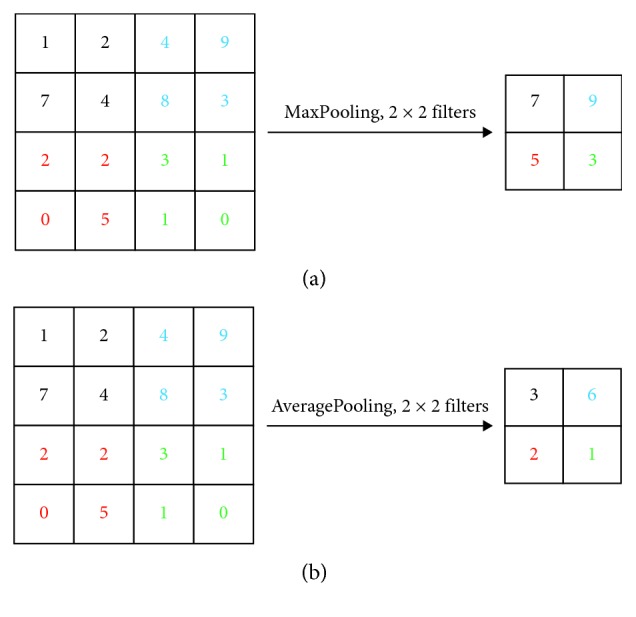
The pooling method. (a) MaxPooling and (b) AveragePooling.

The pooling layer is also called downsampling layer, which follows convolution layer as usual. The error of feature extraction mainly comes from two aspects: (1) the convolutional layer parameter error causes an offset of the estimated mean and (2) the variance of the estimated value caused by the limited size of the neighborhood increases. In pooling processing, the maximum pooling operation (MaxPooling) in Figure 5(b) considers the information around the maximum to be useless, so the information is deleted. MaxPooling can reduce the first error and retain more details. The size of the filters is set to 2 × 2 here. Similar to the average pooling operation (AveragePooling) in Figure 5(a), the data in the filters are averaged to improve the classification of the overall data. AveragePooling can reduce the second error, more background information of the reserved object.

In general, AveragePooling emphasizes a one-level downsampling of the overall feature information. AveragePooling is a bit larger in reducing the contribution of the parameter dimension and more in the dimension of the complete transmission of information. The effect of MaxPooling is better. Although MaxPooling and AveragePooling both sampled the data, MaxPooling felt more like a feature choice. MaxPooling selects features with better classification and provides nonlinearity. At the same time, when MaxPooling reduces the dimension, it is more beneficial to pass the information to the next module for feature extraction. The modulation classification process is like image processing. In the modulation classification process, not all the information comes from the transmitted symbols that need to be identified. There is peripheral information that can be discarded like image processing.

**Figure 6:**
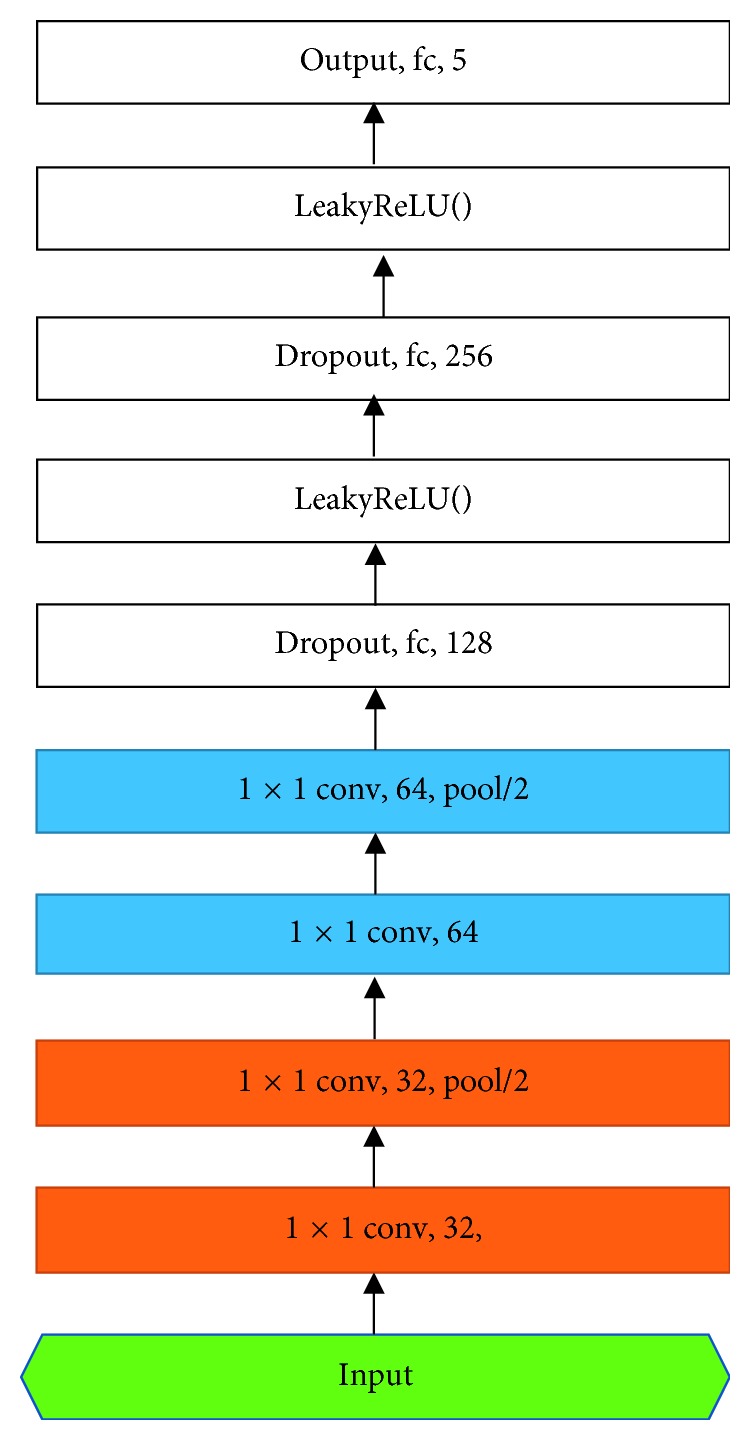
The DL architecture for modulation classification.

The DL architecture is shown in Figure 6, composed of the input player, the hidden layer, and the full connecting layer.

In Figure 6, conv is the convolution, pool/2 is the max pooling 2 × 2 filter, and fc is the fully connected layer. The numbers in each layer represent the number of neurons in each layer, and 1 × 1 represents the size of the filter.

Feature extraction is completed by the middle 4 hidden layer. The first two hidden layers are also known as Conv: 32 × 1 × 1 in Figure 6, as are other hidden layers. The 3 and 4 convolution layer is set to the size of 64 × 1 × 1. In the case of the first hidden layer, Conv: 32 × 1 × 1 represents the layer convolution layer that is made up of 32 neurons and 1 × 1 filters. In the 3-layer and 4-layer hidden layer structure, the intermediate layer selects the form of 64 × 1 × 1 and does not select the form of 32 × 1 × 1. Intuitively, more neurons will have a better fitting effect, the 64 neuron network structure can achieve higher accuracy than the 32 neuron network structure. However, in order to prevent overfitting, no more neurons were selected. The convolutional layer extracts the previous layer of information output to the next layer by designing the convolution core size shown in Figure 6.

The definition of the activation function is as follows:

(7) $$\begin{array}{c} Z_{n}=\varrho \left(w_{n}^{j}\;⊗\;C_{n}+\alpha_{n}^{j}\right), \end{array}$$

where *C*_*n*_ and *Z*_*n*_ are the input and output feature mappings in the *n*th layer, *w*_*n*_^*j*^ and *α*_*n*_^*j*^ are, respectively, the weights and deviations of the *j*th convolution kernel in the nth layer (*n*=1,2,…, 8), *ϱ*(·) is the activation function ReLU, and the operator ⊗ means convolution.

The fully connected layer can be used to map the final output to a linearly separable space. Further, the modulation classification is performed from the learned characteristics of the preceding convolutional part. The fully connected layer consists of two dense layers with Dense 128 and 256 and an output layer with Dense 5. The dense layer with Dense 32 and 64 contains 32 and 64 neurons, and the output layer with Dense 5 contains 5 neurons corresponding to 5 possible modulations, including BPSK, QPSK, 8PSK, 16QAM, and 64QAM. All hidden layers also use ReLU as an activation function. The last output layer uses the Softmax function as an activation function.

ReLU function is defined as follows:

(8) $$\begin{array}{c} g\left(x\right)=\max\left(0,x\right)=\left\{\begin{array}{c} x,\;x\geq 0,\\ 0,\;x<0, \end{array}\right. \end{array}$$

where *x* is the input and *g*(*x*) is the activation function result after the max operation.

The essence of the Softmax function is to map a k-dimensional arbitrary real vector into another k-dimensional real vector, where each element in the vector is between (0,1). Softmax function is defined as follows:

(9) $$\begin{array}{c} \rho {\left(z\right)}_{m}=\frac{e^{z_{m}}}{\sum_{k=1}^{K}e^{z_{k}}}, \end{array}$$

where *z*_*m*_ is the input of the *m*th neuron, *ρ*(*z*)_*m*_ is the output of the *m*th neuron, ∑_*k*=1_^*K*^*e*^*z*_*k*_^ is the sum of the input of all neurons in this layer, and *K* is the number of neurons.

In order to improve performance, the LeakReLU layer is added after the fully connected layer. LeakReLU is a special version of the rectified linear unit. When deactivated, LeakReLU still has a nonzero output value, resulting in a small gradient that avoids the possible neuronal inactivation of the ReLU. LeakReLU function is defined as follows:

(10) $$\begin{array}{c} f\left(x\right)=\left\{\begin{array}{c} x,\;x\geq 0,\\ \alpha x,\;x<0, \end{array}\right. \end{array}$$

where *α* is a floating point number greater than 0, representing the slope of the activation function.

### 3.2. Parameter Settings

During CNN fitting features, if the settings are incorrect, overfitting can easily occur, which result in extremely poor performance of the model when it is actually used. To avoid overfitting, we use dropout technology [27]. Dropout refers to a neural network unit that is temporarily dropped from the network with a certain probability during the training of the deep learning network. After cross validation, the effect is best when the dropout rate of the hidden node is equal to 0.5. The reason is that, at 0.5, the dropout randomly generates the most network structure. Therefore, the dropout factor is set to 0.5 in this paper.

In the training phase, the categorical cross entropy is used as the loss function in the output dense layer [28]. The cross entropy describes the distance between the actual output and the desired output. The smaller the cross entropy, the closer the two probability distributions are. Assuming that the probability distribution *p*(*θ*) is the expected output, the probability distribution *q*(*θ*) is the actual output, *θ* is the sample variables in the sample space, and *H*(*p*, *q*) is the cross entropy, the following equation is obtained:

(11) $$\begin{array}{c} H\left(p,q\right)=-\underset{\theta }{\sum }p\left(\theta \right)\log\;\;q\left(\theta \right). \end{array}$$

The cross entropy reflects the degree of similarity between the distributions *p*(*θ*) and *q*(*θ*).

The choice of the size of the input data batch when training the neural network first determines the direction of the gradient drop. If the dataset is small, it can be in the form of a full dataset. There are at least 2 benefits to doing this: (1) since the gradient values of different weights are greatly different, it is easy to select a global learning rate and (2) the direction determined by the full data set can better represent the overall sample, thus moving more accurately towards the extreme value. For larger data sets, the above two advantages have turned into two disadvantages: (1) iterative in the way of the global learning rate, due to the sampling differences between the various data batches, the gradient correction values are offset against each other and cannot be corrected and (2) the data set generally used to train the network model contains a very large amount of data, and the hardware resources used for network model training, including computer memory and graphics memory capacity, are limited. It is not possible to enter data from all data sets into the network model for training at a time. Since the full dataset does not apply to large data sets, the other extreme is to train only one sample at a time. This is online learning. Using online learning, each correction direction is corrected by the gradient direction of the respective samples, and the traverse is directly inconsistent, and it is difficult to achieve convergence, resulting in the model not being able to train effective results. A better approach is to randomly divide the data into several data blocks of uniform size and then enter them in batches. Compared with the one-time training model, batch training can make the applicability of the model better. If the distribution of the dataset is sufficient, then the gradient calculated with half (or even much less) of the dataset is almost the same as the gradient trained with all the data. The meaning of the gradient is the slope, which is used in machine learning to find the best result (the minimum value of the curve).

Gradient allows us to find the right weight *w*_*n*_^*j*^ and bias *α*_*n*_^*j*^ in formula (7), so that all the training input for the *C*_*n*_ output is approximate to *Z*_*n*_. To measure the extent of the results that are currently being obtained from the goal result, define a cost function:

(12) $$\begin{array}{c} D\left(w,\alpha \right)=\frac{1}{2\lambda }\underset{k}{\sum }\left\|r\left(k\right)-\varepsilon^{2}\right\|, \end{array}$$

where *w* is the set of all weights, *α* is all biases, *λ* is the number of training data, and *ε* is *k* as the input of the output layer vector, summed for all training input *k*. The sign ‖⋯‖ refers to the norm of the vector. *D* is also called the quadratic cost function; sometimes, it is called the squared error or mean squared error (MSE). If the learning algorithm can find the appropriate weights and biases to make *D*(*w*, *α*) ≈ 0, then this is a good learning algorithm. Therefore, the goal of our training algorithm is to minimize the cost function *D*(*w*, *α*) by adjusting the weight and bias of the function. We will use an algorithm called the gradient descent method to achieve this goal.

When adjusting the way the model updates weights and bias parameters, the optimization algorithm can make the model produce better and faster results, such as gradient descent, stochastic gradient descent, and adaptive moment estimation (Adam) [29]. In practical applications, the Adam method works well, and it is able to calculate the adaptive learning rate for each parameter. Compared with other adaptive learning rate algorithms, the Adam convergence speed is faster, the learning effect is more effective, and the problems in other optimization techniques can be corrected, such as the disappearance of the learning rate, the slow convergence. A better performance of the Adam method is that it not only stores the exponential attenuation average of the previous squared gradient but also maintains the exponential attenuation average of the previous gradient.

**Figure 7:**
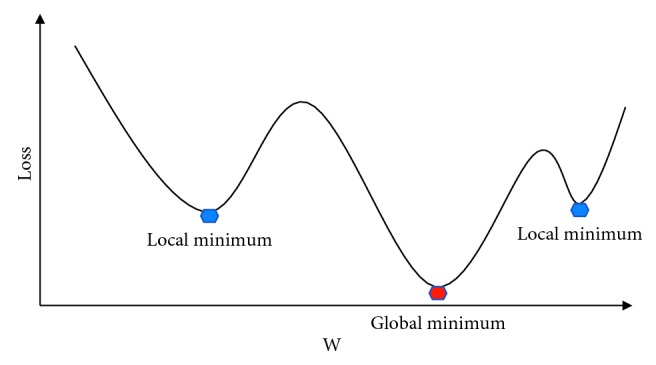
Local minimum diagram.

Too big a batch size tends to get stuck in the sharp minima, resulting in poor generalization when applied after training the model, so it is important to choose a proper size for the batch size [30]. Sharp minima is similar to local minima, indicating that the real global minimum point is not found during the training process, so the network model cannot fully learn the probability distribution of the data set. The local minimum is not the actual minimum point of the whole in Figure 7, but it is easy to fall into the local minimum (represented in blue) during the training process and cannot jump out. In Figure 7, the vertical axis loss represents the global loss and the horizontal axis w represents the weight. The gradient of the solution function, when the gradient value is 0, it can be said that the point is the extreme point of the function. If the function is a convex function, the extreme point is the most significant point. But the multilayer neural network is not a convex function, so theoretically, there are multiple local minimum values, and with the increase in the number of layers, the more local minimum values, local minimum values need to be optimized, such as the batch size selection and dropout technology.

Generalization is the performance of the parameters that the machine learning model learns when it is in the process of learning that the model has not met the sample. Because the computer hardware restriction does not allow you to enter all data sets at once, select a different batch size to be as close to the training accuracy as the full dataset input. The dataset size represents the entire amount of data to be entered into the model, and the batch size represents part of the data in the dataset size. In the case where conditions allow, the entire dataset can be trained in the input model; but in general, limited hardware conditions do not meet such conditions, so the large dataset is divided into the batch input model for training. The batch size will determine the number of samples to be trained at a time and will affect the optimization and speed of the model. The memory efficiency to consider is the choice of a reasonable batch size based on the size of the computer memory and the model training accuracy requirement. The correct choice of the batch size is to find the best balance between the model training accuracy and memory efficiency.

## 4. Simulation and Results

In the simulations experiment, the channel models have been verified by the actual experiment in shadow sea [31]. The channel model parameters in the shallow sea are as follows: the wind speed is 20 knots and the distance between the transmitter and the receiver is 5000 m under the 10 m water. The channel bandwidth is 2 kHz, the carrier frequency is 10 kHz, and the transmit data rate is 1000 symbol/s. The added noise is the Gaussian white noise with band-limited and zero mean. The “white” means a constant power spectrum, and the “Gaussian” refers to the probability that the amplitude takes various values is a Gaussian function. The noise varies with the signal-to-noise ratio (SNR), and the standard deviation is calculated by formula 10^(−SNR/10)^, where the SNR indicates the value of the SNR. The value ranges from −20 dB to 20 dB. The modulation methods of simulation include BPSK, QPSK, 8PSK, and 16QAM used in underwater acoustic communication [8]. In order to further verify the validity of the model, the 64QAM modulation method was added for identification and verification.

Other parameter descriptions are as follows: the batch size is 64. When a complete dataset passes through a neural network once and returns once, the process is called an epoch. However, when an epoch is too large for a computer, it needs to be broken into multiple batches. If a train loss is found to be no drop from the previous epoch training, the training is stopped after 5 epochs. This process, called early stopping, focuses on the model's error on the validation set and stops training when the error does not improve significantly for optimization and related training. Through training data, the train loss is the calculation result of the loss function defined as the categorical cross entropy.

Usually, 50% of the total data is train data and the other 50% is test data, and the validation data are set to the input and output of the test data.

**Figure 8:**
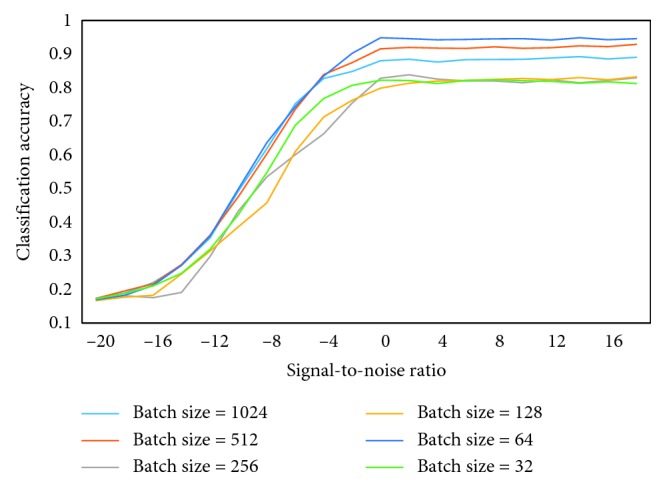
The different batch sizes of classification accuracy.

Different batch sizes sent to the deep network will eventually lead to different recognition rates in Figure 8. Since the final convergence precision will eventually fall into different local extremums, selecting different batch size will achieve the best final convergence accuracy. The batch size = 1 represents a corresponding sample, also called a vector. The batch size represents the number of vectors, the batch size = 64 is 64 vectors. The training size selected in this paper is 64, and the highest precision can be achieved with batch size = 64. When the batch size is of other value, the classification accuracy is not as high as batch size = 64. It shows that different batch size will affect the training effect of the model. For normal data sets, if the batch size is too small, the training data will be very difficult to converge; when the batch size = 32, performance is poor.

**Figure 9:**
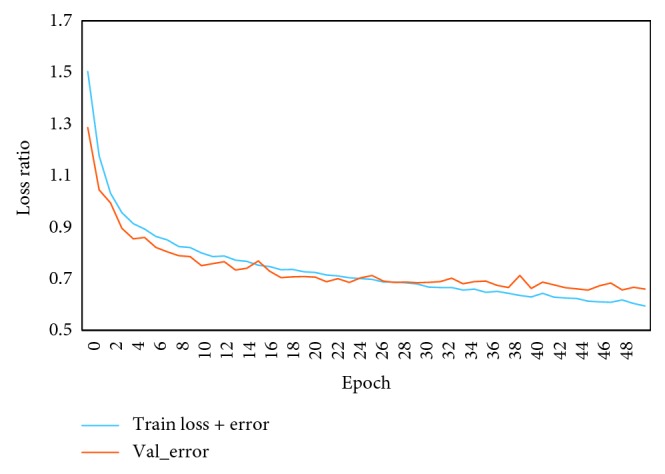
Data training performance.

Only the model with the best performance on the validation set is saved, and the train loss + error value and val_error are minimized in Figure 9. The longitudinal axis loss ratio represents the calculated result of the loss function using formula (11). The training loss + error is the loss function previously defined in the final output layer of the network structure. In other words, train loss + error represents the loss between the predicted data distribution and the actual data distribution resulting from the use of the loss function (the categorical cross-entropy function) in a dataset using a training network model. Here, the loss function [32] is the categorical cross-entropy function defined in equation (11). The val_error is used to predict validation data by using a trained model and verify the predictive effect of the model on the validation set after each epoch. The val_error is similar to the train loss + error, both calculated by the loss function, but they use different data sets. The val_error uses the validation dataset. The minimum val_error value is selected, and the corresponding weight parameter is saved. Finally, these parameters are used to predict test data. At almost 30 epochs, the model has started to converge. Although val_error also fluctuates, it almost converges with the loss of train to a loss rate of about 0.7. The lower number of epochs can reach convergence, it illustrating the effectiveness of our model in such modulation classification tasks.

**Figure 10:**
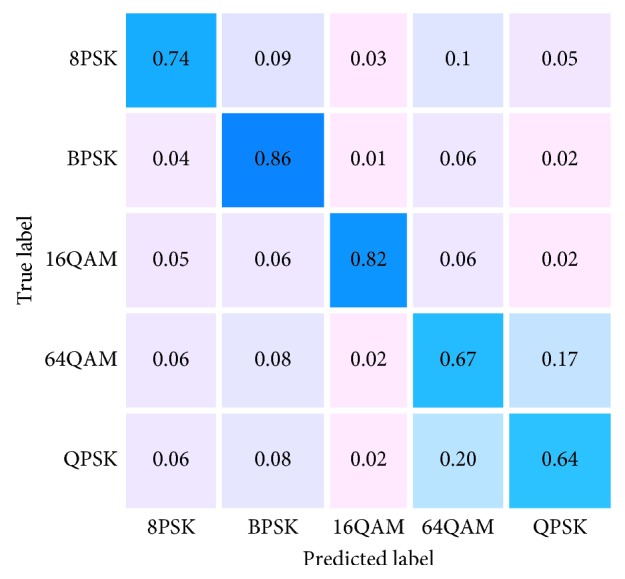
The classification effect of all SNR = −20 dB to SNR = 20 dB.

Figure 10 is the sum of all modulation classification result values from −20 dB to 20 dB, and the classification effect represents the final result of modulation classification (where QAM16 stands for 16QAM and QAM64 stands for 64QAM). The total amount of data in five modulation modes used for training is 50000, and the amount of data for each type of modulation is 10000. After 10, training takes the average. Taking 64QAM as an example, the longitudinal axis is the actual modulation mode, the horizontal axis is the predicted modulation, and the horizontal and longitudinal axis of the intersection in 64QAM is accurate prediction; the darker the color indicates the higher the prediction success rate. The lighter the color, the lower the probability of being misjudged, and the light pink represents almost no misjudgment. When the amount of data is different in Figure 10, DL model shows the basic stability, and 5 modulation methods can be identified, such as MPSK (BPSK, QPSK, and 8PSK) and MQAM (16QAM and 64QAM). From the overall performance point of view, when the data volume is different, the two types of QAM performance difference are obvious. A detailed analysis of the differences in the classification results due to the different amount of quantities will be performed from the specific SNR.

**Figure 11:**
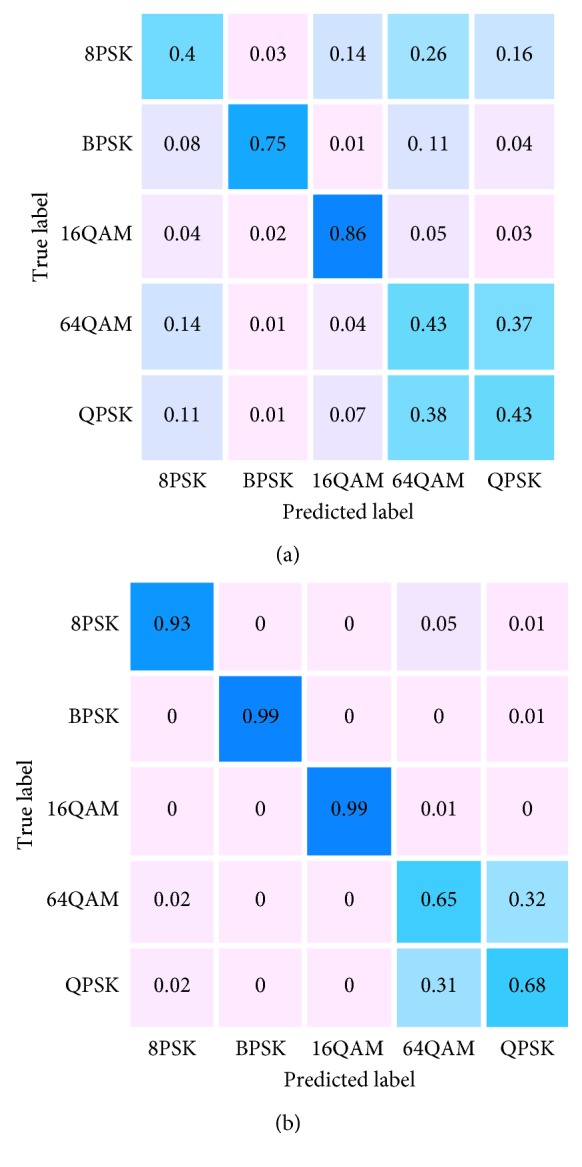
The classification effect. (a) SNR = −10 dB and (b) SNR = −4 dB.

When the low SNR is SNR = −10 dB, the recognition rate of BPSK and 16QAM is relatively high, which can be correctly identified. Other modulation methods are difficult to identify. When the SNR rises to SNR = −4 dB in Figure 10, 8PSK, BPSK, and 16QAM can be almost accurately identified, but the 64QAM and QPSK modulation methods are not easy to distinguish, but the recognition rate has increased by more than 10% when the ratio is SNR = −10 dB in Figure 11.

**Figure 12:**
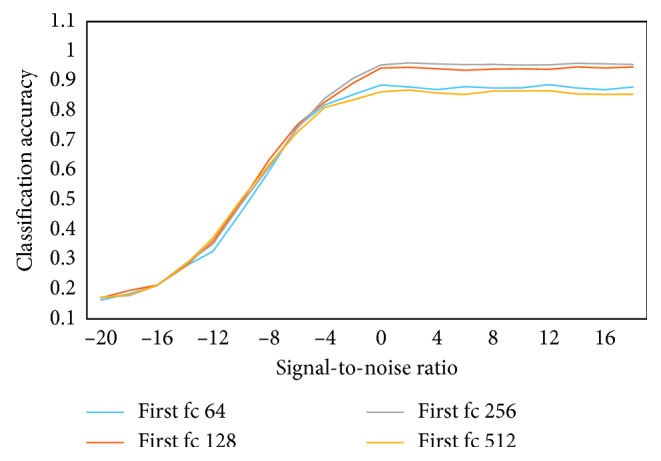
Classification accuracy under different first fc.

In Figure 12, because the increase of the first layer neurons of fc can cause the increase of network model parameters, the parameters of the first layer fc are reduced as far as possible without affecting the performance. Here, in the case of multiple neurons fc layer and similar performance of a small number of neurons fc layer, using a small number of neurons fc layer can reduce the number of parameters, reduce computational complexity, and reduce memory use. First fc under different neuron numbers, the number of corresponding parameters is shown in Table 1. When the first fc is 128 and 256, the performance is almost equivalent, but 128 to 256 parameter is reduced by nearly 2 times. In order to better balance the computational complexity and accuracy of the model, fc is chosen to be 128 as the number of neural units of the model the first fc.

**Figure 13:**
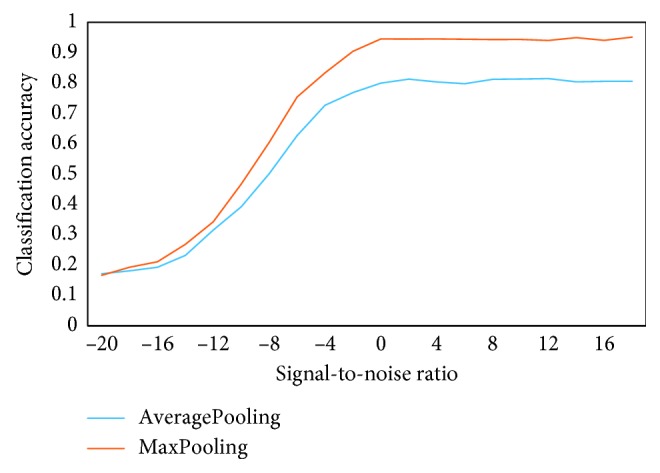
Recognition accuracy under different pooling.

Pooling mainly includes AveragePooling and MaxPooling. The filter size is set to 1 × 1 during AveragePooling; otherwise, the network model cannot converge. In MaxPooling, the filter size is set to 2 *∗* 2 , which can get better performance than AveragePooling. In Figure 13, the performance difference between the two pooling can be more clearly seen.

**Figure 14:**
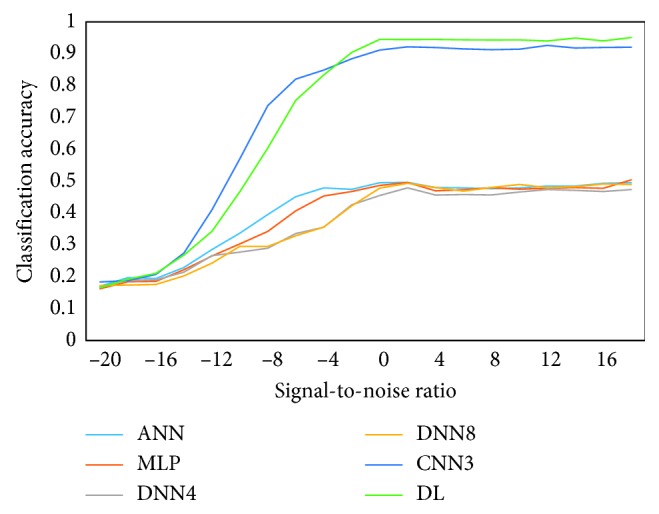
The recognition accuracy for different neural networks.

Figure 14 shows the modulation recognition results using different DL methods, including conventional DL methods such as ANN (artificial neural network) [33], MLP (multilayer perceptron) [34], 4-layer DNN (deep neural networks) and 8-layer DNN [35], CNN (convolutional neural networks) [36], and DL methods used in this article. CNN3 represents a 3-layer CNN network. When the SNR is −20 dB to −15 dB, various neural network recognition capabilities are equivalent, and CNN and DL methods have a slight advantage. When the SNR rises to −15 dB to −5 dB, CNN and DL have obvious advantages over other forms of neural networks, and CNN has a slight advantage over DL in this signal-to-noise ratio range. When the SNR exceeds −5 dB, the DL method used in this paper has a higher recognition rate for CNN, showing the advantages of deep network architecture.

## 5. Conclusions

In this paper, the common modulation methods of underwater communication are effectively recognized by using the deep learning method of the CNN structure. The recognition advantage of the CNN deep neural network structure was discovered. It is not difficult to see from this paper that the deep learning method of CNN form is applicable not only to the field of image recognition but also to the underwater communication field. It is believed that this efficient deep learning method can be more widely applied to signal recognition scenarios for underwater communication. In the future, it will play a greater role in the field of military noncooperative communication and civil adaptive communication. At the same time, it should be noted that, in the case of low SNR of underwater communication, the recognition effect still has room for improvement. In future research, it is necessary to further find a deep learning method of modulation recognition suitable for the low SNR in underwater communication.

## Figures and Tables

**Table 1:** The parameters of first fc under different neuron numbers.

Serial number	Neuron numbers	Total parameters
1	64	4,416,550
2	128	8,823,653
3	256	17,638,373
4	512	35,268,070

## Data Availability

The ∗.dat data used to support the findings of this study have been deposited in the “Dataset for Modulation Classification of Underwater Communication With Deep Learning Network” repository https://drive.google.com/drive/folders/1zZQ0Lo7jEDJXkAMaHAZO1J_--F_PhO8D.
